# PK/PD Analysis of Marbofloxacin by Monte Carlo Simulation against *Mycoplasma* *agalactiae* in Plasma and Milk of Lactating Goats after IV, SC and SC-Long Acting Formulations Administration

**DOI:** 10.3390/ani11041104

**Published:** 2021-04-12

**Authors:** Emilio Fernández-Varón, Edgar García-Romero, Juan M. Serrano-Rodríguez, Carlos M. Cárceles, Ana García-Galán, Carlos Cárceles-García, Rocío Fernández, Cristina Muñoz, Christian de la Fe

**Affiliations:** 1 Center for Biomedical Research (CIBM), Department of Pharmacology, University of Granada, 18071 Granada, Spain; emiliofv@ugr.es; 2 Instituto de Investigación Biosanitaria de Granada (ibs.GRANADA), 18012 Granada, Spain; 3 Department of Animal Health, Faculty of Veterinary Sciences, Regional Campus of International Excellence “Campus Mare Nostrum”, Campus de Espinardo s/n., University of Murcia, 30100 Murcia, Spain; edgar.garcia@um.es (E.G.-R.); ana.garcia25@um.es (A.G.-G.); cdelafe@um.es (C.d.l.F.); 4 Department of Nursing, Pharmacology and Physiotherapy, Pharmacology Area, Faculty of Veterinary Medicine, Universidad de Córdoba, 14071 Córdoba, Spain; RocioFernandezPOC@gmail.com; 5 Department of Pharmacology, Faculty of Veterinary Medicine, Campus de Espinardo s/n., University of Murcia, 30100 Murcia, Spain; carceles@um.es (C.M.C.); carloscarceles@gmail.com (C.C.-G.); 6 Spanish Agency of Medicines and Medical Devices (AEMPS), Parque Empresarial Las Mercedes, 28022 Madrid, Spain; cmunoz@aemps.es

**Keywords:** marbofloxacin, pharmacokinetic, long-acting formulation, lactating goats, contagious agalactia, *M. agalactiae*, Monte Carlo Simulation

## Abstract

**Simple Summary:**

In some countries like Spain and France, contagious agalactia (CA) is a highly relevant issue. CA is a mycoplasmosis affecting small ruminants and it is associated with a relevant economic impact on dairy. The poor efficacy of vaccines and their inability to prevent disease transmission is conducive to the use of antibiotics to control CA. However, only a few groups of antimicrobial agents are effective against these species, and selecting an adequate antimicrobial agent following the categorization of antibiotics made by the different international organisms (European Medicine Agency, World Health Organization) in veterinary medicine becomes a difficult task. The PK/PD approach is a useful tool to guide veterinarians on the appropriate targets through a rational selection of the best dose regimen of antimicrobial agents. In this study, marbofloxacin pharmacokinetics was studied after three routes of administration with two long-acting formulations. The minimum inhibitory concentrations (MIC) values of *Mycoplasma agalactia* isolated from goats affected by CA in Spain were calculated. The results show that systemic exposure achieved in lactating goats following these formulations provides rate of drug release that could be adequate to maintain effective plasma concentrations against *M. agalactiae*. The PK/PD analysis by Monte Carlo simulation showed that a dosage regimen from 8.47 to 11.57 mg/kg every 24 h could effectively treat goats affected by CA.

**Abstract:**

Contagious agalactia is a mycoplasmosis affecting small ruminants that have become an important issue in many countries. However, PK/PD studies of antibiotics to treat this problem in lactating goats affected by *Mycoplasma (M.) agalactiae*, the main CA-causing mycoplasma are almost non-existent. The aims of this study were to evaluate the plasma and milk disposition of marbofloxacin in lactating goats after intravenous (IV), subcutaneous (SC) and subcutaneous poloxamer P407 formulations with and without carboxy-methylcellulose (SC-P407-CMC and SC-P407) administration. Marbofloxacin concentrations were analysed by the High Performance Liquid Chromatography (HPLC) method. Minimum inhibitory concentrations (MIC) of *M. agalactiae* field isolates from mastitic goat’s milk were used to calculate surrogate markers of efficacy. Terminal half-lives of marbofloxacin after IV, SC, SC-P407 and SC-P407-CMC administration were 7.12, 6.57, 13.92 and 12.19 h in plasma, and the half-lives of elimination of marbofloxacin in milk were 7.22, 7.16, 9.30 and 7.74 h after IV, SC, SC-P407 and SC-P407-CMC administration, respectively. Marbofloxacin penetration from the blood into the milk was extensive, with Area Under the Curve (AUC_milk_/AUC_plasma_) ratios ranged 1.04–1.23, and maximum concentrations (C_max-milk_/C_max-plasma_) ratios ranged 0.72–1.20. The PK/PD surrogate markers of efficacy fAUC24/MIC and the Monte Carlo simulation show that marbofloxacin ratio (fAUC_24_/MIC > 125) using a 90% of target attainment rate (TAR) need a dose regimen between 8.4 mg/kg (SC) and 11.57 mg/kg (P407CMC) and should be adequate to treat contagious agalactia in lactating goats.

## 1. Introduction

In the Mediterranean region, the problem of contagious agalactia (CA) is a highly relevant issue. CA is a mycoplasmosis affecting small ruminants. It is associated with a significant economic impact on dairy industries due to its reduction or suppression of milk production and high morbidity rates [1]. CA is mainly produced by *Mycoplasma* (sub)species: *M. agalactiae, M. mycoides subsp. capri, M. capricolum subsp. capricolum and M. putrefaciens* [2]. In some countries like Spain, *M. agalactiae* is more frequently isolated from ovine and caprine flocks with CA [3]. In France, it is becoming an issue, spreading from the Western Pyrenees to other regions [4]. The poor efficacy of vaccines and their inability to prevent disease transmission or infection [5] has initiated the use of antibiotics to control CA [6]. However, some special properties of mycoplasmas, such as lack of a cell wall, drastically reduce the number of antibiotics suitable to be used to treat this issue in small ruminants. Moreover, trying to select an adequate antimicrobial agent following categorization of antibiotics made by the different international organisms (EMA, WHO) in veterinary medicine becomes a difficult task. Only a few groups of antimicrobial agents are effective against these species: tetracyclines, macrolides and fluoroquinolones.

The categorization of the use of antibiotics in the European Union has established four different categories, from A (avoid) to D (prudence). The B (restrict) category includes one of the most effective antibiotic groups to treat mycoplasmosis, fluoroquinolones, which should only be used based on the results of antibiotic susceptibility testing (whenever possible) for the treatment of clinical conditions with no alternative in a lower category [7]. However, several studies have shown that highest resistances of the main veterinary *Mycoplasma* species, including *M. agalactiae* for macrolides, followed by tetracyclines with fluoroquinolones, as the leading alternative treatment [8].

Marbofloxacin is a fluoroquinolone antimicrobial agent extensively used in veterinary medicine with high activity against gram-positive and gram-negative bacteria, including *Mycoplasma *spp. [9,10]. Like other fluoroquinolones, this antimicrobial is a concentration-dependent bactericidal agent and it acts by inhibiting bacterial DNA topoisomerases II and IV [11]. Marbofloxacin is licensed in the EU for use in cattle, including lactating dairy cattle and pigs for treatment of respiratory disease and mastitis, and their maximum residue limits are well known in these species [12]. However, the extra-label use of marbofloxacin may be extended to other species, such as sheep and goats, where the studies are limited. 

Pharmacokinetics of marbofloxacin in non-lactating goats was reported in several studies [13,14,15,16,17]. The influence of endotoxin-induced fever in goats after intravenous administration of marbofloxacin at 2 mg/kg in comparison with normal animals has been described [18]. The same group reported age-related changes in the pharmacokinetics of marbofloxacin after intravenous administration in goats aged 1, 3 and 6 weeks at the same dose level [19]. There are different studies reporting the pharmacokinetics of marbofloxacin in non-lactating species as dogs, sheep, cows and horses [20,21,22,23,24,25], and lactating species as cows, sheep and sows [26,27,28]. However, up until now, the only study reporting pharmacokinetics and milk penetration of marbofloxacin in lactating goats were reported after intravenous and intramuscular administration. This involved a PK/PD study against coagulase negative staphylococci [29] and no studies have been reported for marbofloxacin in lactating goats after SC and SC-long acting formulations. There have been no PK/PD studies of marbofloxacin in lactating goats against *M. agalactiae*, even though it is the most important arising pathogen in many countries causing CA.

P407 is a copolymer that contains 7% polyoxyethylene units and 30% polyoxypropylene blocks. This polymer has some advantages, such as its low toxicity, excellent compatibility with other chemicals and high solubilizing capacity for different drugs. P407 shows temperature-dependent gelation (gel consistency at 37 °C and liquid consistency at 4 °C) [30]. The P407 gel was evaluated for the delivery of antimicrobials such as ceftiofur [30], moxifloxacin [31] and difloxacin [32]. The advantages of the P407 poloxamer formulations in lactating goats might include an increased treatment efficacy (less fluctuations of the stationary concentrations and could have much longer release times) and a reduction in handling (stress of the animals and veterinary costs are decreased). 

The pharmacokinetics and pharmacodynamics (PK/PD) approach is a useful tool to guide veterinarians on the appropriate targets through a rational selection of the best dose regimen of antimicrobial agents [33,34,35,36]. The antibacterial activity of the fluoroquinolones is dependent on the drug concentration and the MIC of the bacterium [37], and the fluoroquinolones exhibit concentration-dependent killing [38]. For that reason, the PK/PD index selected to compute a breakpoint defined as a surrogate of clinical efficacy is the ratio of the Area Under the plasma time Curve (AUC) for free drug to MIC, expressed as fAUC/MIC, when f represents the free or unbound plasma protein drug concentration because only “free antibacterial drug” has antimicrobial activity [39,40].

The selection of a PK/PD index is useful to achieve a target value able to predict clinical efficacy [41]. For fluoroquinolones, typical values for fAUC/MIC ratios are close to 100–125 for gram-negative and 30–50 for gram-positive pathogens have been described [39]. 

Over the last 20 years, information about reliable testing standards has been widely published for the different (classical) microorganisms, including PK-PD principles [42]. However, there is very little reported information about mycoplasmas; no MIC breakpoints for mycoplasmas have been established, and only a few studies reported MIC values against *M. agalactiae* from small ruminants for the small group of antibiotics that can be used to treat these infections [43]. Moreover, no quality control strains are currently available for MIC assays with animal mycoplasmas, and no interpretation criteria are available to be used under the PK/PD approach, mainly due to the problem of the fastidious nature of mycoplasmas to be cultured; thus, only a few laboratories are able to isolate animal mycoplasmas [8]. To our knowledge, this is the first paper to study a PK/PD research of marbofloxacin against *M. agalactiae*.

Thus, the objectives of the present study were: (1) To establish the plasma concentration–time profile and to derive pharmacokinetics data for marbofloxacin in lactating goats after IV, SC, SC-P407 and SC-P407-CMC formulations; (2) To determine the rate and extent of marbofloxacin penetration into and elimination from milk after IV, SC, SC-P407 and SC-P407-CMC administrations; (3) To determine MIC values of *M. agalactia* isolated from goats affected by CA in Spain, and (4) To calculate the PK/PD surrogate markers of efficacy against *M. agalactiae* strains isolated from mastitic goat´s milk and to make an analysis by Monte Carlo simulation from PK parameters of marbofloxacin by IV, SC, SC-P407 and SC-P407-CMC routes to evaluate the efficacy and to propose a dosage regimen.

## 2. Materials and Methods

### 2.1. Animals 

Six clinically healthy Murciano-Granadina female goats weighing between 49.6–68.4 kg and aged from 2.5–3.5 years from the Caprine Farm of the University of Murcia were used. The animals were housed and fed an antibiotic-free diet for at least 30 days preceding the study. For each treatment period of the cross-over, they were observed daily for general health, and clinical observations were made prior to injection and at 2, 10 and 24 h post-injection. Alfalfa hay and water were provided ad libitum together with a drug-free concentrate.

### 2.2. Experimental Design

A cross-over design (latin square 4 × 4) was used in four phases. Each animal received either a single IV or SC injection of marbofloxacin (Marbocyl 10%, Vetoquinol, Madrid, Spain) at a dose of 2 mg/kg or a SC-P407 and SC-P407-CMC administration at a dose quantity of 6 mg/kg with at least a 15-day washout period. 

For the IV administration, the solution was injected into the left jugular vein and blood samples (4 mL) were collected from the contralateral jugular vein into heparinized tubes, SC, SC-P407 and SC-P407-CMC injections were administered under the skin of the back at a single location in the thoraco-lumbar region lateral of the mid-line. Blood samples were collected at 0 (pre-treatment), 0.083, 0.167, 0.25, 0.5, 0.75, 1, 1.5, 2, 4, 6, 8, 10, 12, 24, 32, 48, 72 and 96 h post-dosing. Samples were centrifuged at 1500× *g* for 15 min and the plasma was taken and stored at −90 °C until assayed.

Milk samples for analysis were collected from homogenized milking yields collected immediately before dosing on the day of treatment administration (time 0) and at 1, 2, 4, 6, 8, 10, 12, 24, 32, 48, 72 and 96 h after administration. Milk samples were taken after the complete evacuation of the udder by manual stripping of each gland. For each sample, two aliquots of 5 mL were stored at −90 °C until assayed. 

### 2.3. Long-Acting Formulation Preparation

Gel was prepared on a weight basis using the cold method [44] modified by us. Concentrations of P407 (BASF, Barcelona, Spain), carboxy-methylcellulose (Sigma-Aldrich, Madrid, Spain), and marbofloxacin (Vetranal, Fluka analytical, Sigma-Aldrich, Madrid, Spain) reported here are expressed as weight percentage (% wt/wt). The amount of P407 sufficient to yield 25% gel and carboxy-methylcellulose to yield 2% (in the case of P407-CMC gel) were slowly added to cold water (5 °C) whilst maintaining constant stirring. The dispersion was refrigerated until a clear solution was formed (6–12 h). Marbofloxacin sufficient to yield a 5% concentration was then dissolved in the cold solution.

### 2.4. Analytical Method

Plasma and milk concentrations of marbofloxacin and enrofloxacin as internal standards (Vetranal, Fluka analytical, Sigma-Aldrich, Madrid, Spain) were measured using a modified HPLC method previously reported [45] with fluorescence detection. 

Marbofloxacin and enrofloxacin pure substance was used for quality controls. After the addition of 200 μL of acetonitrile to 200 μL of plasma or milk, the proteins were precipitated by shaking in an ultrasonic bath followed by centrifugation for 10 min at 1600× *g*. The supernatant was diluted fourfold with 0.067 M disodium hydrogen phosphate buffer pH = 7.5 and transferred to HPLC autosampler vials. The HPLC separation was performed using a reverse-phase AscentisTM C18 column (150 × 4.6 mm; 5 μm) with an injection volume of 30 μL. Autosampler vials and column temperature were set at 24 °C. The mobile phase consisted of acetonitrile (20%) and trifluoroacetic acid (1 g/L) (80%) using an isocratic method with a flow rate of 1.0 mL/min. The fluorescence detection was performed at an excitation wavelength of 297 nm and an emission wavelength of 515 nm.

For the method validation, quality controls were prepared from a pool of blank goat plasma or milk spiked with nine concentrations of marbofloxacin and enrofloxacin between 2–2000 µg/L. Linear calibration curves were obtained, and the correlation coefficients (r) were >0.99%. The mean percentage recoveries of marbofloxacin from plasma and milk were 91.17 ± 1.26 and 97.30 ± 1.59%. The limits of quantification (LOQ) and detection (LOD) for plasma and milk were 2 µg/L and 1 µg/L, respectively. The intra-day precision (R.S.D.) was lower than 3% and 4% for plasma and milk, respectively. The inter-day precision (R.S.D.) was lower than 8% and 6% for plasma and milk, respectively. When plasma or milk samples were found to contain a drug concentration greater than the upper limit of the calibration curve (2000 µg/L), they were reanalyzed after dilution with blank plasma or goat milk. Only drug concentrations higher or equal to LOQ were analyzed by pharmacokinetics methods.

### 2.5. MICs Determination

A total of 30 *M. agalactiae* isolates recovered from individual mastitis samples in goat flocks during 2019 and 2020 were selected. Isolates from previously cloned single colonies were identified by PCR [46]. The minimal inhibitory concentration was determined according to the recommendations of Hannan [47]. Briefly, a stationary-phase culture of each isolate was carried out in mycoplasma medium without antimicrobials, supplemented with sodium pyruvate (0.5%) and phenol red (0.005%) in 96-well round-bottomed plates. Marbofloxacin (Tokio Chemical Industry, Tokio, Japan) was added to achieve each of the pre-established final concentrations (from 32 µg/mL to 0.0625 µg/mL) and a final concentration of the mycoplasma cultures of 105 to 103 color-changing units/mL [48]. Positive (lacking marbofloxacin) and negative (lacking mycoplasmas) controls were also added. The plates were then sealed and incubated at 37 °C. After 48 h, plates were examined for color change. MIC was defined as the lowest concentration of marbofloxacin at which no *M. agalactiae* growth (no color change) was observed.

### 2.6. Pharmacokinetic Analysis

Plasma and milk concentration-time curves obtained after each treatment were analysed with PKanalix software version 2020R1 using a non-compartmental approach (Antony, France: Lixoft SAS, 2020). The maximum plasma or milk concentration (C_max_) and the time to reach C_max_ (T_max_) were taken directly from the data. The terminal phase rate constant λ_z_ was estimated by linear regression of time vs. log concentration data, and its terminal half-life as t_1/2λz_ = (ln2)/λ_Z_. The area under the concentration-time curve (AUC), and the first moment time curve (AUMC) for plasma or milk were calculated using the linear-log trapezoidal rule with extrapolation to time infinity. Partial areas were calculated as the area under the concentration-time curve from zero to 24 h (AUC_24_) in plasma and milk. Mean Residence Time was calculated as MRT = AUMC/AUC for both fluids. In the case of IV administration, the systemic plasma clearance was estimated as Cl = Dose/AUC and the apparent volume of distribution at steady state was calculated as V_ss_ = (Dose × MRT)/AUC. For extravascular routes, two parameters were calculated; the mean absorption time as MAT = MRT_(Extravascular routes)_ − MRT_IV_, and the bioavailability (F%) (Equation (1)) by the method of corresponding areas:

(1) $$\left(\%\right)=\frac{{\mathrm{AUC}}_{\left(\mathrm{Extravascular}\;\mathrm{routes}\right)}\times {\mathrm{Dose}}_{\mathrm{IV}}}{{\mathrm{AUC}}_{\mathrm{IV}}\times {\mathrm{Dose}}_{\left(\mathrm{Extravascular}\;\mathrm{routes}\right)}}\times 100$$

In the case of milk, the marbofloxacin concentration at each sampling time interval and the volume of milk at each time interval were used to calculate the percentage of recovery of marbofloxacin in milk.

A binding assay in plasma, or milk, was determined by in vitro equilibrium dialysis. Blank plasma or milk were spiked with marbofloxacin stock solution to obtain final concentrations of 0.025, 0.05, 0.25, 0.50 and 1.00 μg/mL. The assays were performed in a semipermeable membrane (Spectra/Por, molecular weight cut off: 12–14 kDa). Dialysis was performed against sodium phosphate buffer at 64 mM adjusted at pH 7.4 from plasma and pH 6.6 from milk samples. After 24 h at 37 °C with shaking at 100 rpm, samples from both sides were collected and measured by the HPLC method previously described. The unbound fraction (fu) was estimated as the ratio of concentration determined in the buffer with respect to plasma or milk.

### 2.7. PK/PD Analysis 

The surrogate ratios AUC_24_/MIC_50_ and AUC_24_/MIC_90_ in plasma and milk were calculated after PK analysis and MIC determination, and expressed as unbound fraction fAUC_24_/MIC. Moreover, a PK/PD analysis was performed using Monte Carlo simulations in Oracle Crystal Ball V.11.1.1.0.00 software (Oracle Corporation, Redwood Shores, CA, USA). To evaluate the PK/PD cut-off, fAUC_24_ data in plasma or milk on steady-state conditions were combined with MIC data determined of *M. agalactiae* isolates, and included in a 50000-subject simulation [29]. For interpretations of results, several endpoints of PK/PD parameters were used. However, to our knowledge, it is necessary to note that cut-off values for mycoplasmas are not established [4]. From this, fAUC24/MIC values close to 125 from gram-negative bacteria, and between 30–50 from gram-positive bacteria were used as a reference [39]. On the other hand, values obtained experimentally with the surrogate ratios AUC_24_/MIC_50_ and AUC_24_/MIC_90_ were also included.

Data simulated were used to obtain the probability of target attainment or PTA%, defined as the percentage of animals for which the cut-off value of the AUC/MIC index could be attained for different MICs values [40]. The highest probability selected for each PK/PD breakpoint was established as 90% [49].

Monte Carlo simulation was also used to estimate a dose-distribution profile with the PK/PD cut-off values selected combined with the MIC distribution values obtained of *M. agalactiae*. From this, Equation (2) was used:

(2) $$\mathrm{Dose}=\mathrm{Cl}\times \frac{\left(\frac{{\mathrm{AUC}}_{24}}{\mathrm{MIC}}\right){\;x\;\mathrm{MIC}}_{\mathrm{distribution}}}{F\times \mathrm{fu}}$$

where Cl is the total body clearance expressed per hours, AUC_24_/MIC is the PK/PD endpoint, MIC_distribution_ are the values from the distribution of MIC obtained, F is the SC bioavailability, and f_u_ is the unbound fraction of marbofloxacin. However, this equation was modified after the transformation of the PK/PD index by a scaling factor (SF). This value was obtained by dividing the breakpoint AUC_24_/MIC by 24 h. Obviously, plasma clearance was expressed per day and Equation 3 was used to compute the dose [50].

(3) $$\mathrm{Dose}\;\left(\mathrm{per}\;\mathrm{day}\right)={\mathrm{Cl}}_{\mathrm{per}\;\mathrm{day}\;}x\;\frac{\mathrm{SF}\times {\mathrm{MIC}}_{\mathrm{distribution}}}{F\times \mathrm{fu}}$$

Calculations were performed after 50,000-subject Monte Carlo simulations showing the probability of attaining a selected PK/PD breakpoint with different marbofloxacin doses [29].

### 2.8. Statistical Analysis

Normality was assessed by the Kolmogorov–Smirnov test. Data were non-normally distributed and, therefore, non-parametric tests were used. Parameters were represented as the median value with minimum and maximum values. The dose-dependent parameters (C_max_, AUC, AUC_24_ in plasma and milk, and the cumulative amount in milk) were corrected with dose and included in the statistical comparison. The significance level was set at *p* < 0.05. In order to assess the differences between formulations, a statistical comparison has been made by a Kruskal–Wallis test. When significant differences were found, a Wilcoxon rank-sum test was used as a second test to investigate for significant differences between parameters. The statistical software used was Statgraphics Centurion XVI.I for Windows (StatPoint Technologies, Warrenton, VA, USA). Figures were computed using SigmaPlot software (SigmaPlot for Windows version 11.0, 2008, Systat Software Inc, San José, CA, USA).

## 3. Results

Clinical examination of all goats after each phase of the trial did not reveal any abnormalities. No local or systemic adverse reactions were observed after IV, SC, SC-P407 and SC-P407-CMC administration, respectively.

### 3.1. Pharmacokinetic Analysis

**Figure 1:**
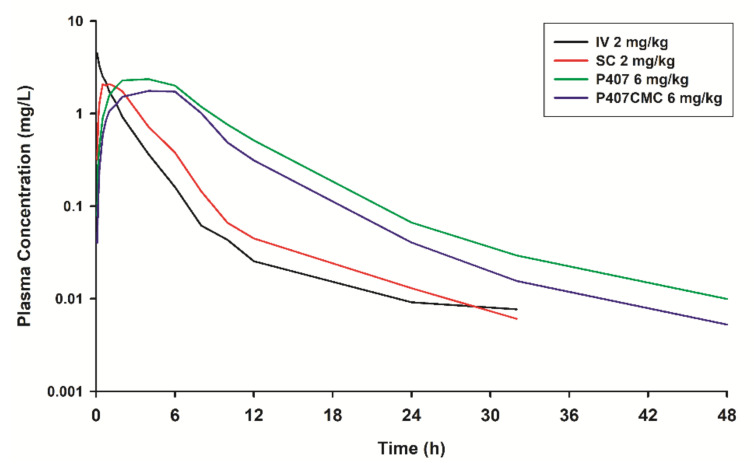
Semilogarithmic plot of plasma concentrations (median) of marbofloxacin after intravenous administration (2 mg/kg) (IV; black line), subcutaneous administration (2 mg/kg) (SC; red line), SC-P407 administration (6 mg/kg) (P407; green line) and SC-P407-CMC administration (6 mg/kg) (P407-CMC; blue line) in goats (*n* = 6).

**Figure 2:**
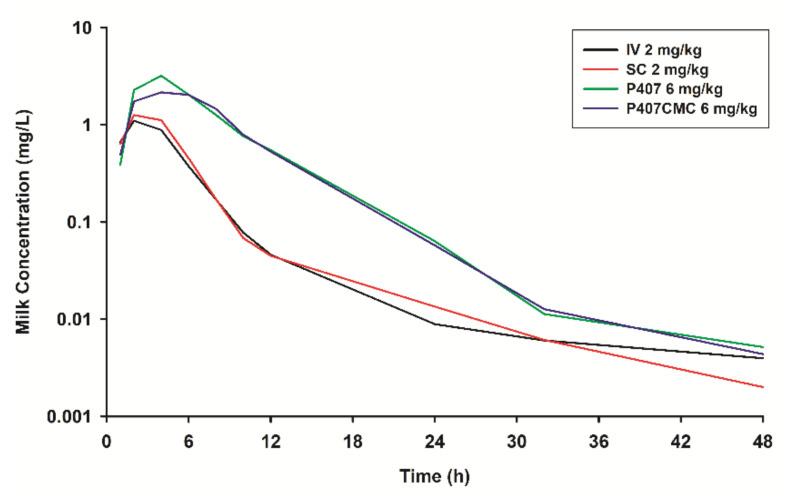
Semilogarithmic plot of milk concentrations (median) of marbofloxacin after intravenous administration (2 mg/kg) (IV; black line), subcutaneous administration (2 mg/kg) (SC; red line), SC-P407 administration (6 mg/kg) (P407; green line) and SC-P407-CMC administration (6 mg/kg) (P407-CMC; blue line) in goats (*n* = 6).

The mean plasma and milk concentrations of marbofloxacin following IV, SC, SC-P407 and SC-P407-CMC administration are plotted in Figure 1 and Figure 2, respectively. 

Median and range values for pharmacokinetic parameters in plasma and milk were calculated for each route of administration and presented in Table 1 and Table 2, respectively.

The marbofloxacin plasma half-life (t½λ_z_) for IV, SC-P407 and SC-P407-CMC routes was 7.12, 6.57, 13.92 and 12.19 h. The clearance value after IV dosing was 0.29 L/kg×h. After SC, SC-P407 and SC-P407-CMC administration, the absolute bioavailability was 93.92% and 103.27% and 81.74%, respectively. The C_max_ was 2.37 mg/L (SC), 2.62 mg/L (SC-P407) and 1.99 mg/L (SC-P407-CMC) with a MAT of 1.54 h (SC), 6.49 h (SC-P407) and 5.35 h SC-P407). Dose-dependent pharmacokinetics parameters from marbofloxacin in plasma and milk after intravenous and extravascular administration normalized to a 2 mg/kg dose are presented in Table 3.

Milk production per day was (mean ± SD) 2.08 ± 0.46 L. The half-lives of elimination of marbofloxacin in milk were 7.22, 7.16, 9.30 and 7.74 h after IV, SC-P407 and SC-P407-CMC administration, respectively. Plasma and milk protein bindings values described as an unbound fraction of marbofloxacin were 0.73 (0.63–0.85) and 0.71 (0.60–0.85), respectively.

### 3.2. M. agalactiae MICs Determination 

The MIC values of marbofloxacin against M. agalactiae (*n* = 30) were obtained and presented in Table 4. Observed MIC values were 0.0625 μg/mL (3.33%), 0.25 μg/mL (46.7%), 0.5 μg/mL (23.3%), 1 μg/mL (20%) and 4 μg/mL (6.7%). MIC_50_ value was 0.25 μg/mL and MIC_90_ was μg/mL. MIC values from the 30 strains tested showed a unimodal distribution with a peak at 0.25 μg/mL.

### 3.3. PK/PD Analysis and Monte Carlo Simulation

**Figure 3:**
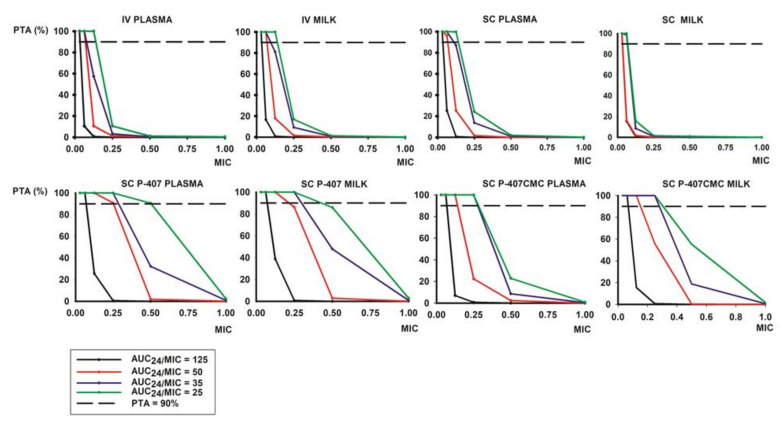
Probability of target attainment (PTA%) values against MIC for each administration route in plasma and milk for AUC/MIC ratio of 125 (black line), AUC/MIC ratio of 50 (red line), AUC/MIC ratio of 30 (blue line) and AUC/MIC ratio of 25 (green line). A PTA% value of 90 is plotted black dashed line.

PK/PD ratios calculated for marbofloxacin against M. agalactiae strains isolated from lactating goats (*n* = 30) are presented in Table 4. Computed dosage per day (mg/kg) based on PK/PD modelling and Monte Carlo simulation to achieve AUC/MIC ratios of marbofloxacin using a 90% of probability of target attainment are shown in Table 5. PTA values of marbofloxacin against M. agalactiae strains using different PK/PD ratios are plotted in Figure 3 where it can be observed that the lowest PTA values are obtained for the highest MICs with all formulations tested. However, better results have been achieved with polymeric formulations, especially when ratios between 25–35, and even 50 for some MIC values. This trend is observed in plasma as well as in milk, indicating that there seems to be no influence between the two fluids.

## 4. Discussion

### 4.1. Pharmacokinetic Analysis

After IV administration, the half-life (t_1/2z_ = 7.12 h) was similar to that reported in non-lactating goats after IV dosing of marbofloxacin (t*_½_*_λ__z_ = 7.18 h) [13] and longer than that reported for lactating goats (3.82 h) [29] and (4.37 h) [16]. However, plasma clearance (Cl = 0.29 L/h·kg) was higher than the data reported by the same authors (0.23 L/h·kg). After SC administration, the terminal half-life (t_1/2λz_ = 6.57 h) in the present study was longer than that reported in non-lactating goats after SC dosing of marbofloxacin (5.74 h) [14] and after intramuscular administration in lactating goats (4.25 h) [29] and no-lactating goats (2.92 h) [17].

The volume of distribution at steady-state (0.97 L/Kg) suggests extensive penetration through biological membranes, and these values indicate that marbofloxacin is widely distributed into the extravascular tissues. This value was close to that reported in lactating goats (1.05 L/kg) [29] and lower than the same parameter in non-lactating goats (1.31 L/kg) [13] and lactating cows (1.5 L/kg) [27], but was higher than the data reported in lactating ewes (0.6 L/kg) [27].

The elimination half-lives (t_1/2λz_) of the SC-P407 (13.92 h) and SC-P407-CMC (12.19 h) formulations were longer than obtained for the IV (7.12 h) and SC administration (6.57 h), showing prolonged permanence of the drug in plasma with these formulations. Mean residence times of the SC-P407 (9.63 h) and SC-P407-CMC (7.69 h) formulations were longer than obtained for the IV (3.05 h) and SC administration (4.04 h). These differences in the plasma terminal half-life and mean residence time, compared to IV and SC formulations, might be due to changes in the rate of drug release and also the administered dose.

The evaluation of marbofloxacin absorption reflect that, probably, the drug was eliminated from plasma after SC-P407 and SC-P407-CMC treatment at a slower rate than after IV and SC treatment, and could be suggesting the presence of a ‘flip-flop’ effect [51] characterized by differences in MAT and MRT_IV_. In fact, when the MAT value for an extravascular formulation is greater than MRT_IV_, the presence of a ‘flip-flop’ effect is suggested [52]. From this approach, the MAT values for SC-P407 (6.49 h) and SC-P407-CMC (5.35 h) were higher than MRT_IV_ (3.05 h), unlike the SC administration (1.54 h). These data suggest that, perhaps, there are not many differences between formulations regardless to the addition of carboxy-methylcellulose at 2% as additive in the P407 gel, also that differences in elimination rate constants and MRT between conventional and poloxamer formulations must be due to differences in the absorption process. In the same way, T_max_ values from polymeric formulations were 3–4 higher than values obtained after SC administration, unlike C_max_, values which were not different despite the use of a higher dose by threefold (Table 1 and Table 3).

Marbofloxacin absorbed well following the administration of SC, SC-P407 and SC-P407-CMC with absolute bioavailabilities (F%) of 93.92%, 103.27% and 81.74%, respectively. Similar values have been obtained for marbofloxacin in goats, sheep and cows [13,27,29] and for other fluoroquinolones in lactating goats [52].

Marbofloxacin is amphoteric due to the presence of a carboxylic acid and one or more basic amine functional groups, being the pKa of 5.69 and 8.02 [11]. At a pH 6–8, this compound is sufficiently lipid-soluble to be able to penetrate the tissues [53]. The penetration of drugs in milk is usually predicted on the basis of the ion trap mechanism [54]. However, various solute transport and secretion processes involved in milk production offer pathways for the movement of drug molecules from plasma to milk [55]. Recently, it was shown that the BCRP protein (also known as ABCG2 efflux protein) is highly expressed in the mammary gland epithelium during lactation, facilitating the excretion of drugs including fluoroquinolones and other xenobiotics into the milk of mice, humans and ruminant species [56,57]. However, in our study, similar concentrations of marbofloxacin in milk compared to plasma concentration were obtained (Figure 1 and Figure 2), despite the ABCG2 gene in goat species has been identified [58]. 

Milk penetration of marbofloxacin occurs in parallel but was slightly lower than that in plasma (Figure 2, Table 2). Indeed, the AUC_milk_/AUC_plasma_ ratios were close to 0.93–1.23, and the C_max-milk_/C_max-plasma_ ratios were close to 0.72–1.20 (Table 2). Similar values of the elimination half-lives (t_1/2λz_) were obtained in milk after IV, SC, SC-P407 and SC-P407-CMC administration (7.22, 7.16, 9.30 and 7.74 h for each treatment respectively) compared to plasma half-lives. Similar results have been obtained for marbofloxacin in lactating goats and cows [28,29]. 

When dose-dependent parameters in plasma and milk were normalized to 2 mg/kg and compared, significantly differences were found in the C_max_ and AUC values from polymeric formulations in relation to SC administration, especially to SC-P407-CMC administration when lower values were observed (Table 3).

### 4.2. MICs Determination and PK/PD Analysis

For fluoroquinolones, AUC_24_/MIC = 100–125 (gram-negative) or 35–50 (gram-positive) ratios have been recommended to achieve high efficacy [33,34,35,36,59,60]. However, these ratios have only been proved effective against these types of bacteria and few studies reported in bibliography with animal mycoplasmas have assumed these ratios as the reference [61,62] This fact is a big limitation to make the PK/PD approach in the present study and although mycoplasmas are presumably evolved by degenerative evolution from gram-positive bacteria, they are phylogenetically most closely related to some clostridia [8] and therefore, we decided to use the AUC_24_/MIC_90_ ratio to estimate the optimum treatment for marbofloxacin in lactating goats affected by CA.

Monte Carlo simulations as a mathematical method employed to calculate the probability of an outcome through repeated random sampling have been used in several fields in science and economics for data analyses and theory confirmation. Monte Carlo simulations are also recommended for analyses of attainment of PK/PD targets to assess antibacterial dosing regimens and determination of PK/PD cutoffs in veterinary medicine [63]. 

The MIC_50_ and MIC_90_ values of marbofloxacin against *M. agalactiae* strains isolated from mastitic goat milk (*n* = 30) were 0.25 and 1 μg/mL, respectively. These values are close to other MICs obtained from field isolates of *M. agalactiae* previously reported [43]. Lower values were observed in ovine strains [4]. Therefore, comparisons should be cautious since the response of the strains may be different between small ruminant species.

Surrogates AUC/MIC_50_ and AUC/MIC_90_ ratios for both plasma and milk were significantly different for the two long-acting formulations with respect to the IV and SC administrations (*p* < 0.05).

The computed dosage based on PK/PD modelling and Monte Carlo simulations using a 90% of PTA (Table 5) were close to 8.5 and 11.5 mg/kg for AUC/MIC index of 125, typically from gram-negative pathogens. However, doses from 3.5 to 4.6 mg/kg and 2.5 to 3.2 mg/kg were obtained using AUC/MIC ratios of 50 and 35 described by gram-positive pathogens. On the other hand, for the lowest AUC/MIC ratio tested close to 25 obtained with our PK and PD data, the computed dosage ranged from 1.7 to 2.3 mg/kg. These data are in agreement with the doses used in this research ranging from 2 to 6 mg/kg, but show that for the two long-acting formulations, no significant differences were found, and no relevant advantage of adding CMC is obtained. All the computed dosage regimens calculated are compatible with the commercial formulations available for other species (cows), as no commercial formulations are registered for goats and extra-label use is needed.

The PTA values against MIC for each administration in plasma and milk from different AUC/MIC ratios are shown in Figure 3. Differences can be observed between IV and SC formulation to the P407 and P407-CMC formulation for each selected index. In fact, for a high index of 125, only the low MIC values of 0.0625 to 0.125 µg/mL could be achieved 90% of PTA in plasma or milk for the four formulations tested (black line into the figure). Moreover, using the index of 50, similar results were observed for IV and SC administrations, whereas for poloxamer formulations, higher MIC values of 0.25 µg/mL could be achieved (red line into the Figure 3). In the same way, with the index of 35, the same trend could be observed for IV and SC dosing in plasma and milk. However, the opposite is observed with the polymeric administrations when MIC values similar or higher than 0.25 µg/mL were achieved (blue line in Figure 3). However, using the low index of 25 obtained with the PK/PD ratios described in Table 4 for MIC_90_ values, higher values of 0.5 µg/mL can be achieved with poloxamer formulations in relation to a 90% of PTA value (green line into the Figure 3).

The PTA values obtained suggest that doses from 2 to 6 mg/kg, as used in this research, would not achieve a positive outcome for a ratio of 125, but could be moderately satisfactory for an index of 50, and more appropriate with ratios between 35 and 25. These last values could be typical of using a gram-positive index as a reference. However, they also indicate that the best results are presented with poloxamer P-407 formulations in all ratios. Moreover, these data also suggest that higher doses could reach the most demanding ratios in a more accurate way, which is in accordance with the doses per day previously calculated (Table 5). In this regard, the differences in PK/PD parameters between SC, SC-P407 and SC-P407-CMC administration are consequences of the threefold SC dose for P407 and P407-CMC formulation. Normalized doses for the four routes studied are shown in Table 3. However, it is necessary to note that the numerical values of AUC_24_/MIC recommended as surrogate markers to predict optimal dosage for concentration-dependent antimicrobial drugs were generated in experimental infections in laboratory animals or in human clinical trials [40]; thus, these numerical values might not be applicable to goat infections or to animal infections in general. This is especially true when mycoplasmas are used as bacterial targets from small ruminant species due to the physiological differences and little knowledge of its cut-off points and PK/PD ratios in comparison with other types of bacteria, such as gram-positive or -negative bacteria [4,43]. In fact, it has been widely reported in ex vivo experiments with time-killing curves where the surrogate markers (AUC/MIC) for ruminant species were lower than 100 [23]. In addition, different factors such as protein binding, tissue distribution, the immunocompetence of the host and the fact that the concentration-dependent killing profile of fluoroquinolones is associated with a relatively prolonged post-antibiotic effect should be considered cautiously with these PK-PD ratios [23].

Furthermore, in lactating goats, we had to take into account that inflammation of the mammary gland leads to vascular permeability changes and differences in milk composition. Milk pH generally increases, milk casein concentrations decrease, milk albumin concentrations increase, somatic cells increase, and milk fat contents can decrease. Therefore, it would be necessary to take many precautions to extrapolate these specific parameters to lactating goats, especially in the case of contagious agalactia, where experiences about the more appropriate values of these parameters do not currently exist.

## 5. Conclusions

In main lines, the systemic exposure achieved in lactating goats following IV, SC and the long-acting formulations SC-P407 and SC-P407-CMC administration of marbofloxacin provides a rate of drug release that could be adequate to maintain effective plasma concentrations for the duration of the dosage interval. In the case of SC-P407 and SC-P407-CMC formulations, high plasma levels are only achieved after 48 h.

The antibacterial drug used in this study allowed a good penetration in milk, with a concentration-time profile in milk closely paralleled the plasma profile.

The MICs determination of *M. agalactiae* field strains and the PK/PD analysis by Monte Carlo simulation show that a dosage regimen from 8.47 to 11.57 mg/kg every 24 h to achieve the most exigent AUC/MIC ratio of 125 would be appropriate for a positive therapeutic outcome. However, lower doses from 1.5 to 4.5 mg/kg could be more effective with AUC/MIC ratios from 25 to 50. In this context, it is necessary to take into account that this is the first research that relates PK/PD parameters with *M. agalactiae*. In addition, these results should be analyzed with caution due to the special characteristics of mycoplasmas, lack of studies and references to approach this issue from the PK/PD approach making necessary further studies to evaluate and optimize the best therapeutic strategies for treating CA in lactating goats.

## Figures and Tables

**Table 1:** Pharmacokinetic parameters (median and range) for marbofloxacin in plasma of lactating goats (*n* = 6) after IV and SC administration at a dose of 2 mg/kg body weight, and as SC-P407 gel and SC-P407-CMC gel formulations at a dose of 6 mg/kg bodyweight.

Parameters	IV	SC	SC-P407	SC-PCMC
λ_z_ (1/h)	0.10 (0.08–0.15)	0.11 (0.09–0.15)	0.05 (0.03–0.08) ^a,b^	0.06 (0.014–0.08) ^a,b^
t_1/2λz_ (h)	7.12 (4.72–9.13)	6.57 (4.68–7.79)	13.92 (8.99–24.42) ^a,b^	12.19 (8.86–17.14) ^a,b^
C_max_ (mg/L)		2.37 (1.04–2.75)	2.62 (1.74–4.06)	1.99 (1.54–2.95)
T_max_ (h)		1.00 (1.00–1.50)	3.00 (2.00–4.00) ^b^	4.00 (2.00–6.00) ^b^
AUC_0-inf_ (mg·h/L)	6.82 (5.53–8.93)	8.86 (4.06–12.00)	21.99 (17.73–27.78)	17.24 (13.47–20.00)
AUC_24_ (mg·h/L)	6.82 (5.53–8.93)	8.74 (3.92–11.71)	20.25 (16.33–26.53)	16.48 (12.95–19.02)
MRT (h)	3.05 (2.20–3.56)	4.04 (3.35–4.87)	9.63 (6.15–10.21) ^a,b^	7.69 (7.07–9. 11) ^a,b^
MAT (h)		1.54 (0.19–1.80)	6.49 (2.59–7.18) ^b^	5.35 (4.25–5.56) ^b^
V_SS_ (L/kg)	0.91 (0.75–1.14)			
Cl (L/h/kg)	0.29 (0.22–0.35)			
F%		93.92 (64.91–113.40)	103.27 (69.19–117.10)	81.74 (68.31–106.47)

λ_z_: the first order rate constant associated with the terminal (log-linear) elimination phase. t_1/2__λz_: the elimination half-life associated with the terminal (log-linear) elimination phase. C_max_: the peak or maximum plasma concentration following extravascular administration. T_max_: the time to reach peak or maximum plasma concentration following extravascular administration. AUC_0_^inf^: the area under the plasma concentration-time curve from zero to infinity. AUC_24_: the area under the plasma concentration-time curve from zero to 24 h. MRT: mean residence time. MAT: mean absorption time. V_ss_: the apparent volume of distribution at steady state. Cl: Plasma clearance. F%: the fraction of the administered dose systemically available (bioavailability). ^a^ Significantly different from IV (*p* < 0.05). ^b^ Significantly different from SC (*p* < 0.05).

**Table 2:** Pharmacokinetic parameters (median and range) for marbofloxacin in the milk of lactating goats (*n* = 6) after IV and SC administration at a dose of 2 mg/kg body weight and as SC-P407 gel and SC-P407-CMC gel formulations at a dose of 6 mg/kg of bodyweight.

Parameters	IV	SC	SC-P407	SC-P407-CMC
λ_z_ (1/h)	0.10 (0.06–0.18)	0.10 (0.07–0.14)	0.07 (0.04–0.12) ^a,b^	0.09 (0.06–0.12) ^a,b^
t_1/2λz_ (h)	7.22 (3.87–11.37)	7.16 (4.83–9.68)	9.30 (86.00–17.44) ^a,b^	7.74 (6.02–11.16) ^a,b^
C_max_ (µg/mL)	1.78 (0.71–2.67)	1.41 (0.85–3.14)	3.26 (1.46–4.63)	2.38 (1.78–2.66)
T_max_ (h)	2.00 (1.00–4.00)	2.00 (2.00–4.00)	4.00 (2.00–6.00) ^a,b^	4.00 (2.00–6.00) ^a,b^
AUC (µg·h/mL)	7.98 (4.45–11.61)	6.96 (4.15–14.08)	23.36 (14.84–35.54)	19.34 (16.09–28.08)
AUC_24_ (µg·h/mL)	7.83 (4.31–11.52)	6.84 (4.05–13.87)	22.89 (14.28–34.92)	18.93 (15.79–26.41)
MRT (h)	4.79 (4.28–5.80)	5.05 (4.62–5.62)	7.73 (6.62–8.68) ^a,b^	7.85 (6.27–10.43) ^a,b^
C_max-milk_/C_max-plasma_		0.72 (0.47–1.51)	1.20 (0.47–1.89)	1.11 (0.90–1.50)
AUC_milk_/AUC_plasma_	1.04 (0.79–1.37)	0.93 (0.72–1.59)	0.95 (0.76–1.68)	1.23 (0.81–1.59)
Recovery (%)	0.76 (0.11–2.69)	0.59 (0.24–1.34)	0.60 (0.51–1.52)	0.52 (0.40–0.76)

λ_z_: the first order rate constant associated with the terminal (log-linear) elimination phase. t½ λz: the elimination half-life associated with the terminal (log-linear) elimination phase. C_max_: the peak or maximum milk concentration following extravascular administration. T_max_: the time to reach peak or maximum milk concentration following extravascular administration. AUC_0-inf_: the area under the milk concentration-time curve from zero to infinity. AUC_24_: the area under the milk concentration-time curve from zero to 24 h. C_max-milk_/C_max-plasma_: milk/plasma máximum concentration ratios. AUC_milk_/AUC_plasma_: milk/plasma máximum area under the curve concentration ratios from zero to infinity. Recovery: percentage excreted in milk. ^a^ Significantly different from IV (*p* < 0.05). ^b^ Significantly different from SC (*p* < 0.05).

**Table 3:** Dose-dependent pharmacokinetic parameters from marbofloxacin in plasma and milk after intravenous and extravascular administration normalised to a 2 mg/kg dose.

Plasma	IV	SC	SC-P407 ^n^	SC-P407-CMC ^n^
C_max_ (mg/L)		2.37 (1.04–2.75)	0.87 (0.58–1.35) ^b^	0.66 (0.51–0.98) ^b^
AUC (mg·h/L)	6.82 (5.53–8.93)	8.86 (4.06–12.00)	7.33 (5.91–9.26)	5.75 (4.49–6.67) ^a,b,c^
AUC_24_ (mg·h/L)	6.82 (5.53–8.93)	8.74 (3.92–11.71)	6.75 (5.44–8.84)	5.49 (4.32–6.34) ^a,b,c^
Milk	IV	SC	SC-P407 ^n^	SC-P407-CMC ^n^
C_max_ (mg/L)	1.78 (0.71–2.67)	1.41 (0.85–3.14)	1.09 (0.49–1.54)	0.79 (0.59–0.89) ^b^
AUC (mg·h/L)	7.98 (4.45–11.61)	6.96 (4.15–14.08)	7.79 (4.95–11.85)	6.45 (5.36–9.36)
AUC_24_ (mg·h/L)	7.83 (4.31–11.52)	6.84 (4.05–13.87)	7.63 (4.76–11.64)	6.31 (5.26–8.80)

Plasma: C_max_: the peak or maximum plasma concentration following extravascular administration. AUC: the area under the plasma concentration-time curve from zero to infinity. AUC_24_: the area under the plasma concentration–time curve from zero to 24 h. Milk: C_max_: the peak or maximum milk concentration. AUC: the area under the milk concentration-time curve from zero to infinity. AUC_24_: the area under the milk concentration–time curve from zero to 24 h. C. amount: the cumulative amount of marbofloxacin eliminated in milk. ^a^ Significantly different from IV (*p* < 0.05). ^b^ Significantly different from SC (*p* < 0.05). ^c^ Significantly different from SC-P407 (*p* < 0.05). ^n^ Dose-dependent parameter corrected to a 2 mg/kg dose.

**Table 4:** PK/PK ratios estimated for marbofloxacin against *M. agalactiae* strains isolated from goats (n = 30), as well as MIC_50_ and MIC_90._

Parameters	IV	SC	SC-P407	SC-P407-CMC
AUC_24_/MIC_50_ (h) plasma	27.27 (22.13–35.70)	34.94 (15.69–46.82)	81.02 (65.33–106.12) ^a^	95.92 (51.79–76.08) ^a^
AUC_24_/MIC_50_ (h) milk	31.32 (17.25–46.08)	27.35 (16.22–55.47)	91.55 (57.12–139.70) ^a^	75.70 (63.15–105.66) ^a^
AUC_24_/MIC_90_ (h) plasma	6.82 (5.53–8.93)	8.74 (3.92–11.71)	20.25 (16.33–26.53) ^a^	16.48 (12.95–19.02) ^a^
AUC_24_/MIC_90_^n^ (h) milk	7.83 (4.31–11.52)	6.84 (4.05–13.87)	22.89 (14.28–34.92) ^a^	18.93 (15.79–26.41) ^a^
MIC_50_ (μg/mL)	0.25	Range (μg/mL) 0.0625–4.00		
MIC_90_ (μg/mL)	1.00			

^a^ Significantly different from IV (*p* < 0.05).

**Table 5:** Computed dosage (mg/kg) based on PK/PD modelling and Monte Carlo simulation to achieve AUC/MIC ratios of marbofloxacin using a 90% of PTA as an endpoint.

	Dosage Calculated to Achieve a 90% PTA of Endpoint			
AUC/MIC Ratio	IV	SC	P407	P407CMC
125	9.65	8.47	8.83	11.57
50	3.85	3.48	3.45	4.61
30	2.81	2.44	2.35	3.2
25	1.96	2.27	1.73	2.3

PTA = Probability of target attainment (probability for the plasma concentration to exceed the endpoint for clinical efficacy).

## Data Availability

The data presented in this study are available on request from the corresponding author.

## References

[1] Prats-van der Ham M., Tatay-Dualde J., Ambroset C., De la Fe C., Tardy F. (2018). The moderate drift towards less tetracycline-susceptible isolates of contagious agalactia causative agents might result from different molecular mechanisms. Vet. Microbiol..

[2] Corrales J.C., Esnal A., De la Fe C., Sanchez A., Assuncao P., Poveda J.B., Contreras A. (2007). Contagious agalactia in small ruminants. Small Rumin. Res..

[3] Ariza-Miguel J., Rodríguez-Lázaro D., Hernández M. (2012). A survey of *Mycoplasma agalactiae* in dairy sheep farms in Spain. BMC Vet. Res..

[4] Poumarat F., Gautier-Bouchardon A.V., Bergonier D., Gay E., Tardy F. (2016). Diversity and variation in antimicrobial susceptibility patterns over time in Mycoplasma agalactiae isolates collected from sheep and goats in France. J. Appl. Microbiol..

[5] Agnone A., La Manna M.P., Loria G.R., Puleio R., Villari S., Nicholas R.A., Guggino G., Sireci G. (2013). Timing of activation of CD4+ memory cells as a possible marker to establish the efficacy of vaccines against contagious agalactia in sheep. Vet. Immunol. Immunopathol..

[6] Gómez-Martín A., Amores J., Paterna A., De la Fe C. (2013). Contagious agalactia due to *Mycoplasma *spp. in small dairy ruminants: Epidemiology and prospects for diagnosis and control. Vet. J..

[7] EMA, Committee for Medicinal Products for Veterinary use (CVMP) (2020). Categorisation of Antibiotics in the European Union. EMA/CVMP/CHMP/682198/2017. https://www.ema.europa.eu/en/news/categorisation-antibiotics-used-animals-promotes-responsible-use-protect-public-animal-health.

[8] Gautier-Bouchardon A.V. (2018). Antimicrobial Resistance in *Mycoplasma* spp.. Antimicrobial Resistance in Bacteria from Livestock and Companion Animals.

[9] Drugeon H., Thomas V., Gaillandeau L., Thomas E. (1997). Antibacterial activity of marbofloxacin against bovine respiratory isolates. In: Proceedings of the 7th EAVPT International Congress, Madrid, Spain. J. Vet. Pharmacol. Ther..

[10] Meunier D., Acar J.F., Martel J.L., Kroemer S., Valle M. (2004). Seven years survey of susceptibility to marbofloxacin of bovine pathogenic strains from eight European countries. Int. J. Antimicrob. Agents..

[11] Martínez M., McDemott P., Walker R. (2006). Pharmacology of the fluorquinolones: A perpective for the use in domestic animals. Vet. J..

[12] EMA (2009). Committee for Veterinary Medicinal Products. Marbofloxacin (Extensión to All Food Producing Species). Summary Report. http://www.ema.europa.eu/docs/en_GB/document_library/Maximum_Residue_Limits_-_Report/2009/11/WC500014864.pdf.

[13] Waxman S., Rodríguez C., González F., De Vicente M.L., San Andrés M.I., San Andrés M.D. (2001). Pharmacokinetic behavior of marbofloxacin after intravenous and intramuscular administrations in adult goats. J. Vet. Pharmacol. Ther..

[14] Dova S.W., San Andrés M.D., González F., San Andrés M.I., De Lucas J.J., Rodríguez C. (2007). Pharmacokinetic behavior and pharmacokinetic/pharmacodynamic integration of marbofloxacin after subcutaneous administration in goats. Vet. J..

[15] Sidhu P.K., Landoni M.F., Aliabadi F.S., Lees P. (2010). Pharmacokinetic and pharmacodynamic modelling of marbofloxacin administered alone and in combination with tolfenamic acid in goats. Vet. J..

[16] Bhardwaj P., Sidhu P.K., Lonare M.K., Kaur R., Dumka V.K., Rampal S. (2018). Pharmacokinetic-pharmacodynamic integration of marbofloxacin after single and repeated intravenous administration in goats. Res. Vet. Sci..

[17] Bhardwaj P., Sidhu P.K., Saini S.P.S., Rampal S. (2019). Pharmacokinetic-pharmacodynamic relationship of marbofloxacin for *Escherichia coli* and *Pasturella multocida* following repeated intramuscular administration in goats. J. Vet. Pharmacol. Ther..

[18] Waxman S., San Andrés M.D., González F., De Lucas J.J., San Andrés M.I., Rodríguez C. (2003). Influence of *Escherichia coli* endotoxin-induced fever on the pharmacokinetic behavior of marbofloxacin after intravenous administration in goats. J. Vet. Pharmacol. Ther..

[19] Waxman S., San Andrés M.D., González F., San Andrés M.I., De Lucas J.J., Rodríguez C. (2004). Age-related changes in the pharmacokinetics of marbofloxacin after intravenous administration in goats. J. Vet. Pharmacol. Ther..

[20] Thomas V., Deleforge J., Boisrame B. (1994). Pharmacokinectics of marbofloxacin in pre-ruminant and ruminant cattle. Sixth EAVPT Congress Proceedings.

[21] Thomas V., Deleforge J., Boisrame B., Espinasse J. (1994). Pharmacokinetics of marbofloxacin in healthy and sick pre-ruminant calves. Sixth EAVPT Congress Proceedings.

[22] Schneider M., Thomas V., Boisrame B., Deleforge J. (1996). Pharmacokinetics of marbofloxacin in dogs after oral and parenteral administration. J. Vet. Pharmacol. Ther..

[23] Aliabadi F.S., Lees P. (2002). Pharmacokinetics and pharmacokinetic/pharmacodynamic integration of marbofloxacin in calf serum, exudate and transudate. J. Vet. Pharmacol. Ther..

[24] Bousquet-Melou A., Bernard S., Schneider M., Toutain P.L. (2002). Pharmacokinetics of marbofloxacin in horses. Equine Vet. J..

[25] Sidhu P.K., Landoni M.F., Aliabadi F.S., Lees P. (2010). PK-PD integration and modeling of marbofloxacin in sheep. Res. Vet. Sci..

[26] Petracca K., Riond J.L., Graser T., Wanner M. (1993). Pharmacokinetics of the gyrase inhibitor marbofloxacin: Influence of pregnancy and lactation in sows. Zentralbl. Veterinarmed. A.

[27] Shem-Tov M., Ziv G., Glickman A., Saran A. (1997). Pharmacokinetics and penetration of marbofloxacin from blood into the milk of cows and ewes. Zentralbl. Veterinarmed. A.

[28] Schneider M., Vallé M., Woehrlé F., Boisramé B. (2004). Pharmacokinetics of marbofloxacin in lactating cows after repeated intramuscular administrations and pharmacodynamics against mastitis isolated strains. J. Dairy Sci..

[29] Lorenzutti A.M., Litterio N.J., Himelfarb M.A., Zarazaga M.D.P., San Andrés M.I., De Lucas J.J. (2017). Pharmacokinetics, milk penetration and PK/PD analysis by Monte Carlo simulation of marbofloxacin, after intravenous and intramuscular administration to lactating goats. J. Vet. Pharmacol. Ther..

[30] Zhang L., Parsons D.L., Navarre C., Kompella U.B. (2002). Development and in-vitro evaluation of sustained release poloxamer 407 (P407) gel formulations of ceftiofur. J. Control Release.

[31] Cárceles C.M., Serrano J.M., Marín P., Escudero E., Fernández-Varón E. (2006). Pharmacokinetics of moxifloxacin in rabbits after intravenous, subcutaneous and a long-acting poloxamer 407 gel formulation administration. J. Vet. Med. A Physiol. Pathol. Clin. Med..

[32] Marín P., Escudero E., Fernández-Varón E., Ramírez M.J., Cárceles C.M. (2010). Pharmacokinetics and milk penetration of difloxacin after a long-acting formulation for subcutaneous administration to lactating goats. J. Dairy Sci..

[33] Ambrose P.G. (2006). Monte Carlo simulation in the evaluation of susceptibility breakpoints: Predicting the future: Insights from the society of infectious diseases pharmacists. Pharmacotherapy.

[34] Craig W.A. (1998). Pharmacokinetic/pharmacodynamic parameters: Rationale for antibacterial dosing of mice and men. Clin. Infect. Dis..

[35] Martinez M.N., Papich M.G., Drusano G.L. (2012). Dosing regimen matters: The importance of early intervention and rapid attainment of the pharmacokinetic/pharmacodynamic target. Antimicrob. Agents Chemother..

[36] Martinez M.N., Toutain P.-L., Turnidge J. (2013). The pharmacodynamics of antimicrobial agents. Antimicrobial Therapy in Veterinary Medicine.

[37] Walker R.D. (2000). Fluoroquinolones. Antimicrobial Therapy in Veterinary Medicine.

[38] McKellar Q.A., Sanchez Bruni S.F., Jones D.G. (2004). Pharmacokinetic/pharmacodynamic relationships of antimicrobial drugs used in veterinary medicine. J. Vet. Pharmacol. Ther..

[39] Papich M.G. (2014). Pharmacokinetic-pharmacodynamic (PK-PD) modeling and the rational selection of dosage regimes for the prudent use of antimicrobial drugs. Vet. Microbiol..

[40] Toutain P.L., Sidhu P.K., Lees P., Rassouli A., Pelligand L. (2019). VetCAST Method for Determination of the Pharmacokinetic-Pharmacodynamic Cut-Off Values of a Long-Acting Formulation of Florfenicol to Support Clinical Breakpoints for Florfenicol Antimicrobial Susceptibility Testing in Cattle. Front. Microbiol..

[41] Toutain P.L., Bousquet-Mélou A., Damborg P., Ferran A.A., Mevius D., Pelligand L., Veldman K.T., Lees P. (2017). En Route towards European Clinical Breakpoints for Veterinary Antimicrobial Susceptibility Testing: A Position Paper Explaining the VetCAST Approach. Front. Microbiol..

[42] Clinical and Laboratory Standards Institute (2019). Understanding Susceptibility Test Data as a Component of Antimicrobial Stewardship in Veterinary Settings.

[43] Tatay-Dualde J., Prats-van der Ham M., de la Fe C., Paterna A., Sánchez A., Corrales J.C., Contreras A., Gómez-Martín A. (2017). Mutations in the quinolone resistance determining region conferring resistance to fluoroquinolones in Mycoplasma agalactiae. Vet. Microbiol..

[44] Schmolka I.R. (1972). Artificial skin. I. Preparation and properties of pluronic F-127 gels for treatment of burns. J. Biomed. Mater. Res..

[45] Siefert H.M., Kohlsdorferc C., Steinkec W., Witt A. (1999). Pharmacokinetics of the 8-methoxyquinolone, moxifloxacin: Tissue distribution in male rats. J. Antimicrob. Chemother..

[46] Marenda M.S., Sagné E., Poumarat F., Citti C. (2005). Suppression subtractive hybridization as a basis to assess Mycoplasma agalactiae and Mycoplasma bovis genomic diversity and species-specific sequences. Microbiol. Read..

[47] Hannan P.C. (2000). Guidelines and recommendations for antimicrobial minimum inhibitory concentration (MIC) testing against veterinary mycoplasma species. International Research Programme on Comparative Mycoplasmology. Vet. Res..

[48] Albers A.C., Fletcher R.D. (1982). Simple method for quantitation of viable mycoplasmas. Appl. Environ. Microbiol..

[49] Asín-Prieto E., Rodríguez-Gascón A., Isla A. (2015). Applications of the pharmacokinetic/pharmacodynamic (PK/PD) analysis of antimicrobial agents. J. Infect. Chemother..

[50] Lees P., Pelligand L., Illambas J., Potter T., Lacroix M., Rycroft A., Toutain P.L. (2015). Pharmacokinetic/pharmacodynamic integration and modelling of amoxicillin for the calf pathogens Mannheimia haemolytica and Pasteurella multocida. J. Vet. Pharmacol. Ther..

[51] Toutain P.L., Bousquet-Mélou A. (2004). Plasma terminal half-life. J. Vet. Pharmacol. Ther..

[52] Toutain P.L., Bousquet-Mélou A. (2004). Bioavailability and its assessment. J. Vet. Pharmacol. Ther..

[53] Fernández-Varón E., Villamayor L., Escudero E., Espuny A., Cárceles C.M. (2006). Pharmacokinetics and milk penetration of moxifloxacin after intravenous and subcutaneous administration to lactating goats. Vet. J..

[54] Brown S.A. (1996). Fluoroquinolones in animal health. J. Vet Pharmacol. Ther..

[55] Atkinson H.C., Begg E.J. (1990). Prediction of drug distribution into human milk from physicochemical characteristics. Clin. Pharmacokinet..

[56] McManaman J.L., Neville M.C. (2003). Mammary physiology and milk secretion. Adv. Drug. Deliv. Rev..

[57] Pulido M.M., Molina A.J., Merino G., Mendoza G., Prieto J.G., Alvarez A.I. (2006). Interaction of enrofloxacin with breast cancer resistance protein (BCRP/ABCG2): Influence of flavonoids and role in milk secretion in sheep. J. Vet. Pharmacol. Ther..

[58] Schrickx J.A., Fink-Gremmels J. (2008). Implications of ABC transporters on the disposition of typical veterinary medicinal products. Eur. J. Pharmacol..

[59] Wu H.J., Luo J., Wu N., Matand K., Zhang L.J., Han X.F., Yang B.J. (2008). Cloning, sequence and functional analysis of goat ATP-binding cassette transporter G2 (ABCG2). Mol. Biotechnol..

[60] AliAbadi F.S., Lees P. (2000). Antibiotic treatment for animals: Effect on bacterial population and dosage regimen optimisation. Int. J. Antimicrob. Agents.

[61] Mitchell J.D., McKellar Q.A., McKeever D.J. (2012). Pharmacodynamics of antimicrobials against Mycoplasma mycoides mycoides small colony, the causative agent of contagious bovine pleuropneumonia. PLoS ONE.

[62] Zhang N., Gu X., Ye X., Wu X., Zhang B., Zhang L., Shen X., Jiang H., Ding H. (2016). The PK/PD Interactions of Doxycycline against Mycoplasma gallisepticum. Front. Microbiol..

[63] Xiao X., Lan W., Zhao Y., Li R., Liu Y., Liu J., Wang Z. (2021). In vivo Pharmacokinetic and Pharmacodynamic (PK/PD) Modeling and Establishment of the PK/PD Cutoff of Florfenicol against Pasteurella multocida in Ducks. Front. Microbiol..

